# Clerodane furanoditerpenoids as the probable cause of toxic hepatitis induced by Tinospora crispa

**DOI:** 10.1038/s41598-018-31815-6

**Published:** 2018-09-10

**Authors:** Xavier Cachet, Jerôme Langrand, Ludivine Riffault-Valois, Chouaha Bouzidi, Cyril Colas, Annabelle Dugay, Sylvie Michel, Denis Boucaud-Maitre

**Affiliations:** 1 Laboratoire de Pharmacognosie, UMR 8638 COMETE CNRS, Faculté de Pharmacie de Paris, Université Paris Descartes, Sorbonne Paris Cité, Paris, F-75006 France; 2 Centre Antipoison de Paris, AP-HP Hôpital Lariboisière-Fernand Widal, Paris, F-75010 France; 3 Institut de Chimie Organique et Analytique, ICOA UMR 7311 CNRS Université d’Orléans, Orléans, F-45071 France; 4 Centre de Biophysique Moléculaire, CBM UPR 4301 CNRS, Orléans, F-45071 France; 5 Dispositif de Toxicovigilance Antilles, Centre Hospitalier de la Basse-Terre, Basse-Terre, F-97100 France; 6 Direction de la Recherche Clinique et de l’Innovation, Centre Hospitalo-Universitaire de Guadeloupe, Pointe-à-Pitre, F-97110 France

## Abstract

*Tinospora crispa* is a popular traditional herbal plant commonly used throughout the world for treatment of various diseases, in particular type 2 diabetes mellitus. We report here a new case of toxic hepatitis in a 57-year old male patient in the French West Indies following the consumption of two aqueous extracts of fresh *Tinospora crispa* stems. It thus differs from two previously reported cases that concerned the chronic intake of powdered dry stems delivered in solid oral dosage forms (*i.e*. pellets and tablets). Liquid Chromatography-Diode Array Detection-Mass Spectrometry (LC/DAD/MS) analyses were performed on an aqueous extract of the offending sample that mimics the swallowed preparation. They revealed the presence of species-specific molecular marker borapetoside C (**1**) and thus enabled an unambiguous phytochemical identification. The exploration of tandem MS/MS data obtained by ultra-high performance liquid chromatography/electrospray ionization quadrupole time-of-flight mass spectrometry (UHPLC-ESI-QTOF-HRMS) allowed the identification of 17 additional *cis*-clerodane-type furanoditerpenoid lactones, analogues of **1**. These results support the hypothesis that the mechanisms underlying hepatotoxicity of *Tinospora crispa* are the same as those encountered with furanoditerpenoids-containing plants such as *Teucrium chamaedrys* or *Dioscorea bulbifera*. In the context of type 2 diabetes treatment, we recommend that *Tinospora crispa* intake should be more closely monitored for signs of hepatotoxicity.

## Introduction

*Tinospora crispa* (L.) Hook. f. & Thomson belongs to the botanical family of Menispermaceae. The plant is widely distributed in primary rain forests in Southeast Asia, China and Africa. It is also present in French Guyana and the French West Indies as an ornamental plant for gardens. It is a popular traditional medicinal plant in Asia, mainly as a stem decoction^1^, for the treatment of diabetes, jaundice, rheumatism, urinary disorders, fever, malaria or hypertension. Its potential for the treatment of diabetes is of interest for the following reasons: the growing number of people with type 2 diabetes in the world, the cost of conventional glucose-lowering medications (and the limitations related to their efficacy on glycaemic control in the long term/run), and the high consumer demand for alternative therapy. Promising results have been recently observed both *in vitro* and *in vivo* by several teams of researchers, raising hope for future application in diabetology^2^. However, conflicting results have been obtained in clinical studies and its safety profile remains to be determined. Indeed, a potential hepatoxicity has been raised by two case reports following the chronic consumption of pellets or tablets containing *T. crispa* in Asia^3,4^. Here we report the new case of a patient having developed toxic hepatitis due to the consumption of two aqueous extracts of *T. crispa* stem in the French West Indies. We discuss the benefit/Risk of the use of this plant as an alternative therapy for the treatment of diabetes in the light of current literature. Liquid Chromatography-Diode Array Detection-Mass Spectrometry (LC/DAD/MS) and Liquid Chromatography-tandem Mass Spectrometry (LC/MS/MS) profiling enabled an unambiguous phytochemical identification of the incriminated sample and revealed the presence of putative toxins, namely the *cis*-clerodane-type furanoditerpenoid lactones in an aqueous stem extract similar to the traditional preparation.

An expeditious targeted qualitative analysis of these secondary metabolites was performed by combining classical LC/MS/MS data exploration (*i.e*. tentative identification of the compounds based on the exact mass and fragmentation pathways/formal identification by comparison of the retention time (t_R_), UltraViolet (UV) spectrum and tandem mass spectrometry (MS/MS) data with a reference compound) and molecular networking analysis (MS^2^-MN)^5,6^. This has allowed to observe possible similarities between compound spectra and thus to study shared fragment ions.

## Case Report

A 57-year-old male patient, with no personal or family history of liver disease or alcohol addiction, was admitted for acute hepatitis with cholestasis and cytolysis. Three weeks before, he had started consuming an aqueous extract of “lian-sepent”, a traditional medicine corresponding to *T. crispa* according to local ethnobotanists. This herbal remedy was supposed to detoxify his liver. It consisted of a piece of *T. crispa* stem put into a bottle of water and drunk regularly over the next two days. Two weeks later, the patient prepared a similar aqueous extract of *T. crispa* and consumed it over two days. Following his last intake, the patient felt fever and asthenia for one week and went to the emergency room after occurrence of dark urine. On admission, he suffered from jaundice, was not overweight, and had normal vital signs. Biological tests revealed evidence of hepatocellular damage (ALT 1923 U/L; AST 873 U/L) and cholestasis (γGT 155 U/L). Abdominal ultrasonography was normal with no hepatomegaly or lithiasis. The results of laboratory testing disclosed no serological arguments for viral hepatitis (hepatitis A virus, hepatitis B virus, hepatitis C virus, cytomegalovirus, Epstein-Barr virus and varicella zoster virus). The patient was discharged two weeks after admission and evolution was marked by the regression of jaundice and progressive decrease in liver function tests without any specific treatment (Table 1).

**Table 1:** Evolution of hepatic laboratory parameters.

Parameter (normal value)	03/03/2016	06/03/2016	10/03/2016	22/03/2016
ALT (15–50 U/L)	1923	1673	1246	215
AST (10–50 U/L)	873	639	480	52
γGT (10–71 U/L)	155	192	181	124
Total bilirubin (1.0–17.1 µmol/L)	Not available	52	46.5	11.9

The first laboratory test was performed one week after the last intake of *T. crispa* decoction.

## Laboratory Investigations

### Identification of plant sample

**Figure 1:**
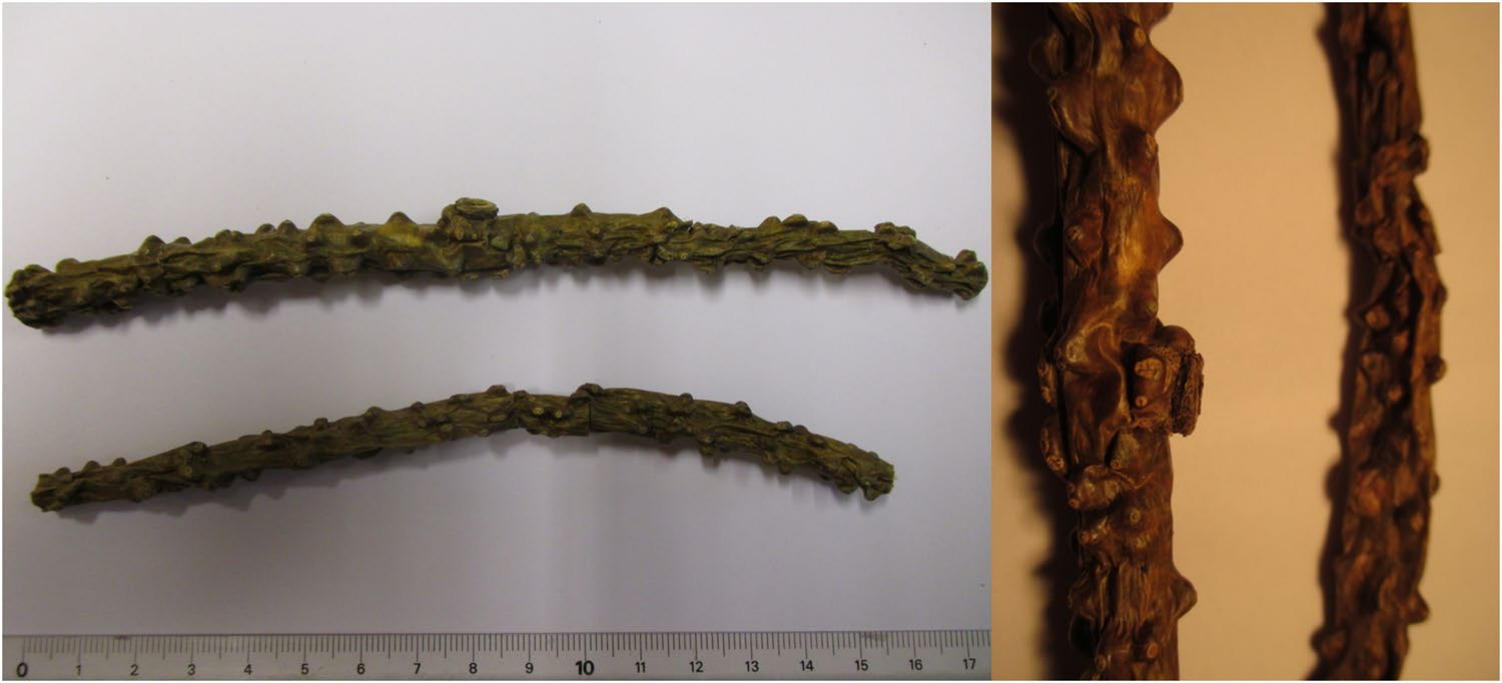
Sample of *Tinospora crispa* involved in toxic hepatitis (Author: X.C.).

The sample provided by the patient consisted of fresh stems of *T. crispa* as ascertained by comparison of both macroscopic botanical features and phytochemical profile, with those of an authentic herbal reference standard^4^. As shown in Fig. 1, stems display a prominent tuberculate surface absent in the widespread related medicinal species *T. cordifolia* (Willd.) Miers and *T. sinensis* (Lour.) Merr. (synonyme of *T. malabarica* (Lam.) Hook f. & Thomson)^7^.

**Figure 2:**
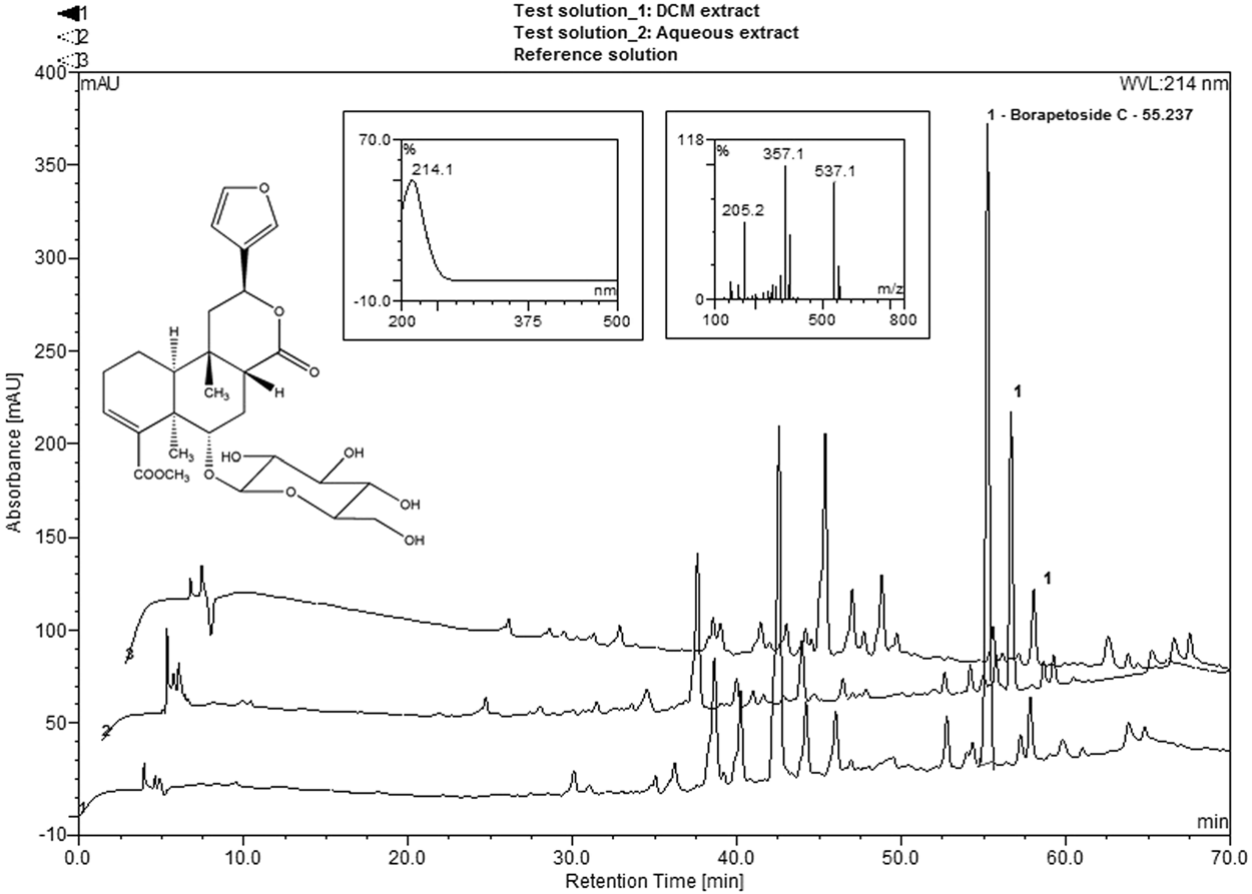
Reverse Phase-HPLC-UV chromatograms of CH_2_Cl_2_ extracts: (lower trace: 1): offending sample, (upper trace: 3): reference sample; of aqueous extract: (middle trace: 2); with an offset of 3 min. Embedded data (from right to left): borapetoside C (**1**): chemical structure, UV/Visible spectrum and positive Electrospray Ionisation-Mass Spectrometry (ESI-MS) spectrum.

Chromatographic profiles of both the analysed and the reference samples were next established by High-Performance Liquid Chromatography with Diode-Array Detection-Mass Spectrometry HPLC-DAD-MS for their corresponding CH_2_Cl_2_ extracts (Fig. 2).

Borapetoside C (**1**)^8,9^ (synonym tinocrisposide), a species-specific molecular marker of *Tinospora crispa* was unambiguously identified in the herbal reference standard as well as in the suspect sample by comparison of the t_R_, UV spectrum and MS data with a reference compound isolated from the plant (t_R_ 55.2 min; λ_max_: 214 nm; *m/z* 559: Na adduct [M + Na]^+^, 537: pseudo-molecular ion [M + H]^+^; fragment ions at *m/z* 375, 357, 339, 325, 311, 307, 297, 279, 251, 205, 187 and 159). Interestingly, this compound appeared as the main peak on the LC-UV (214 nm) chromatogram of the analysed sample (Fig. 2, lower chromatogram). The amount of this compound was clearly found higher in the fresh stems than in the older reference sample, moreover consisting of dried stems (Fig. 2, upper chromatogram).

### Phytochemical analysis of an aqueous extract that mimics the traditional preparation

We next investigated whether borapetoside C (**1**) and other potentially toxic related clerodane furanoditerpenoids could be identified in an aqueous extract of fresh stems that mimics the traditional preparation.

First of all, the peak assigned to **1** was also found predominant in the chromatogram obtained by HPLC/DAD/MS and depicted in Fig. 2 (middle chromatogram). However, this finding was not really surprising since glycosides are essentially water-soluble.

Subsequently, a targeted qualitative analysis of the furanoditerpenoids structurally related to **1** was carried out in two ways: by using ultra-high performance liquid chromatography/electrospray ionization quadrupole time-of-flight mass spectrometry (UHPLC-ESI-QTOF-MS/MS) and by analysing the generated data on the basis of the exact mass of the compounds and their fragmentation pathways, in combination with a molecular networking approach for the whole dataset processing and especially their filtering. For this purpose, the data analysis portal of the Global Natural Products Social Molecular Networking (GNPS) web-based platform (http://gnps.ucsd.edu) was used. This approach constitutes a powerful and innovative tool for the dereplication of natural products and consequently for the identification of new ones. Building of molecular networks is based on relatedness analysis within tandem MS/MS data. It enables to visualize them as a molecular network clustering together data for structurally related molecules. MS/MS spectra are represented as nodes connected together by edges symbolizing close similarities between them^5,6^. Consequently, introducing this step in the workflow allowed an efficient data filtering and facilitated the targeting of compounds structurally analogous to borapetoside C (**1**).

**Figure 3:**
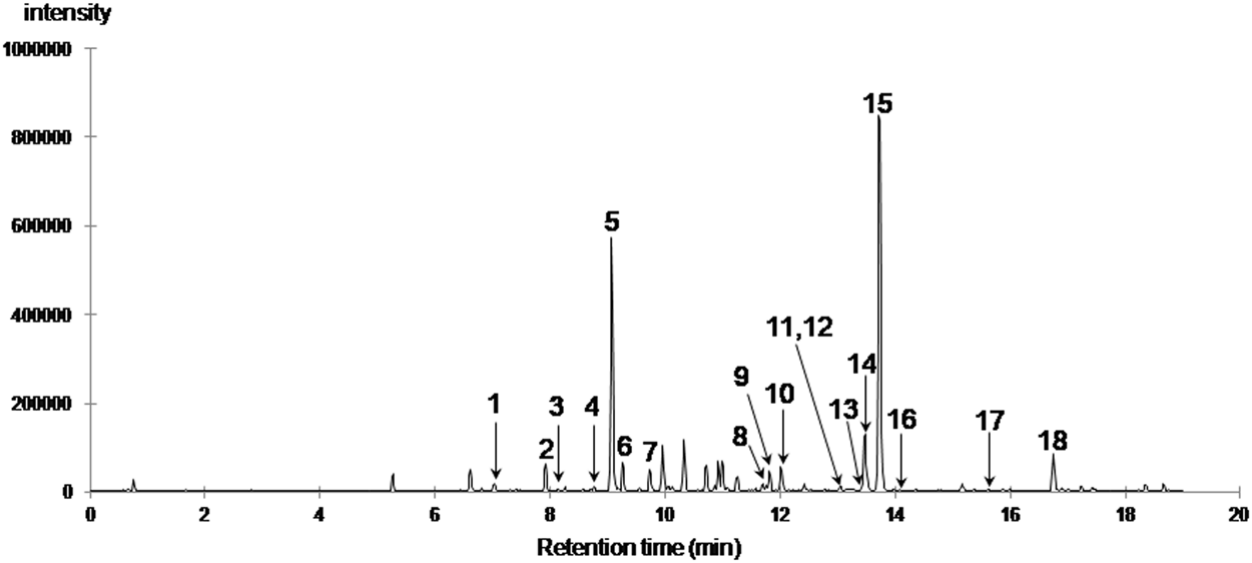
UHPLC-ESI-QTOF-HRMS base peak chromatogram of the *T. crispa* stems aqueous extract (numbered peaks corresponds to the furanoditerpenoids identified in this study).

**Figure 4:**
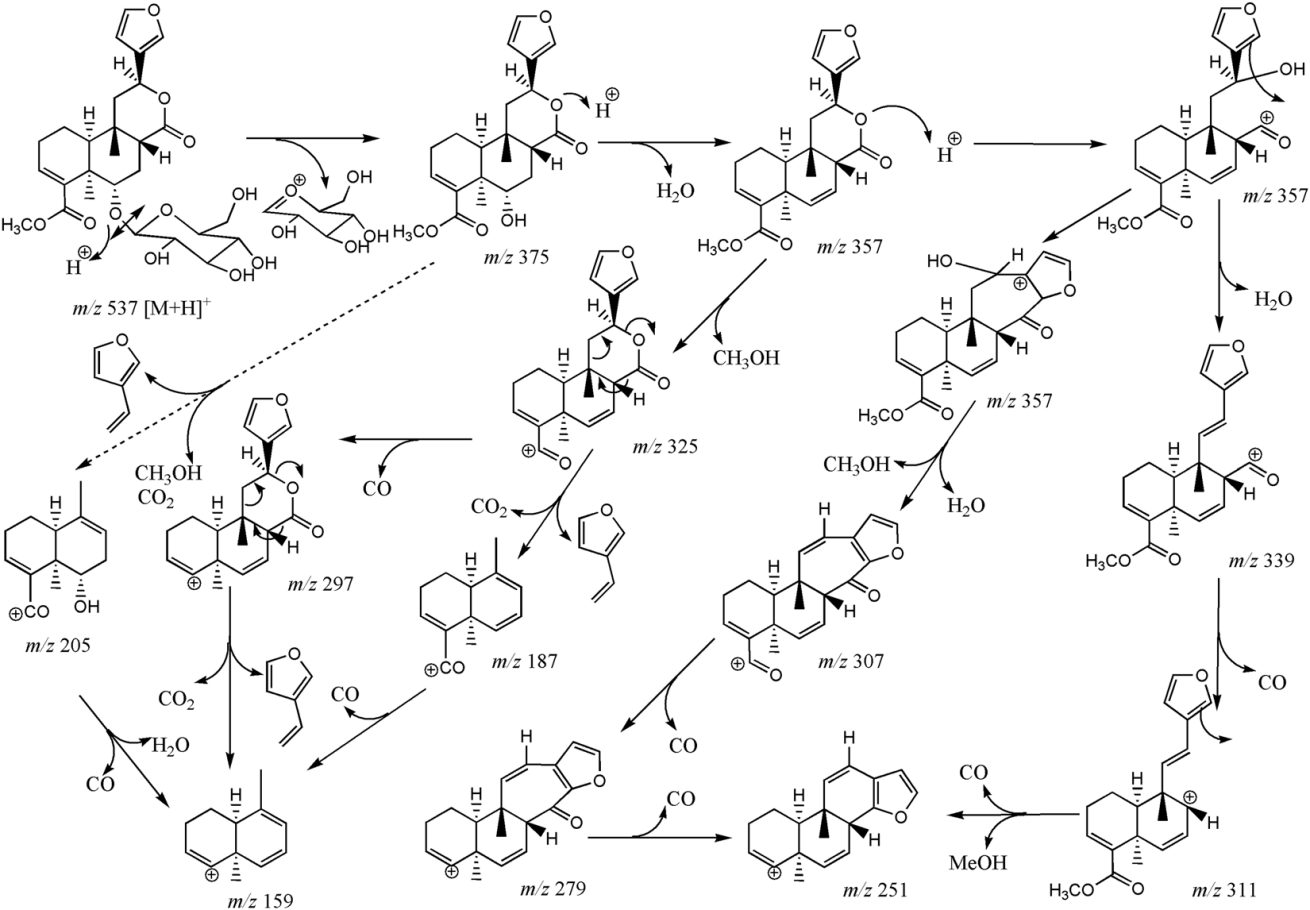
Proposed fragmentation pathways of borapetoside C (**1**).

The chromatographic profile of the aqueous extract obtained by ultra-high performance liquid chromatography/electrospray ionization quadrupole time-of-flight mass spectrometry (UHPLC-ESI-QTOF-HRMS) was found similar to the one previously obtained by HPLC/DAD/MS (see Fig. 2). A representative base peak chromatogram (BPC) with numbered peaks (No. 1–18), corresponding to the furanoditerpenoids detected in this study, is illustrated in Fig. 3. In these conditions, the peak (No. 15) corresponding to borapetoside C (**1**) was detected with a t_R_ of 13.7 min (data matching those observed for standard). Major fragment ions observed in the MS/MS spectra of **1** are listed in Table 2, and the logical fragmentation pathways for this compound are described in Fig. 4.

**Table 2:** Identification of furanoditerpenoids in the aqueous extract of *T. crispa* stems by UHPLC-QTOF/HRMS^2^.

Peak number	*t*_R_ (min)	*m/z*	Molecular formula	Δmass error (ppm)	Ion type	Other detected *m/z* on MS scan (error in ppm, ion type)	ESI-HRMS^2^ data (*m/z*)	Putative* metabolite identification (*except for peak 15 (cpd 1)
1	7.0	715.2828	C_33_H_47_O_17_	2.8	[M + H]^+^	737.2642 (2.0, [M + Na]^+^)	**553.2282**, 341.1404, 323.1326	Borapetoside H
2	7.9	593.2209	C_27_H_38_NaO_13_	−1.2	[M + Na]^+^		**553.2285**, 391.1753, 341.1390, 323.1266	Unknown furano-diterpenoid structurally related to borapetoside B (C_27_H_38_O_13_)
3	8.2	**685.2687**	C_32_H_45_O_16_	−2.2	[M+H]^+^	702.2966 (−0.3, [M+NH4]^+^), 707.2526 (0.6, [M+Na]^+^)	341.1381, 323.1291, 295.1346	Compound **2** or unknown isomer
4	8.7	**685.2700**	C_32_H_45_O_16_	−0.3	[M+H]^+^	702.2980 (1.7, [M+NH4]^+^), 707.2528 (0.9, [M+Na]^+^)	391.1792, 341.1399, 323.1277, 305.1179, 295.1333	Compound **2** or unknown isomer
5	9.1	**553.2277**	C_27_H_37_O_12_	−0.5	[M+H]^+^	**570.2543** (−0.4, [M+NH_4_]^+^), 575.2095 (−0.7, [M+Na]^+^)	391.1748, 341.1375, 323.1278, 305.1170, 295.1334	Borapetoside B or isomer **3** or rumphioside I
6	9.3	**553.2279**	C_27_H_37_O_12_	−0.1	[M+H]^+^		**373.1656**, 341.1389, 323.1283, 295.1335, 267.1377	Borapetoside B or isomer **3** or rumphioside I
7	9.7	545.1998	C_26_H_34_NaO_11_	+0.9	[M+Na]^+^	567.1804 (−1.5, [M-H+2Na]^+^)	383.1468, 365.1358, **343.1541**, 251.1432, 203.0531, 159.0795, 131.0855	Unknown furanoditerpenoid structurally related to **1** (C_26_H_34_O_11_)
8	11.7	383.1465	C_20_H_24_NaO_6_	−0.1	[M+Na]^+^		**343.1543**, 251.1430, 187.0754,159.0799, 131.0856	Crispene B
9	11.8	**699.2842**	C_33_H_47_O_16_	−2.4	[M+H]+	716.3127 (0.4, [M+NH4]^+^), 721.2681 (0.4, [M+Na]^+^)	**357.1708**, 339.1585, 325.1441, 307.1347, 279.1368, 251.1426, 205.0859, 159.0801, 131.0843	Borapetoside D or unknown isomer (C_33_H_46_O_16_)
10	12.0	413.1570	C_21_H_26_NaO_7_	−0.2	[M+Na]^+^	803.3233 (−2.1, [2M+Na]^+^)	**373.1647**, 267.1388, 159.0806, 131.0850	Borapetol B
11	13.0	**699.2859**	C_33_H_47_O_16_	0.0	[M+H]^+^	721.2687 (1.2, [M+Na]^+^)	357.1700, 339.1597, 325.1439, 307.1336, 297.1513, 279.1383, 205.0867, 197.0737, 153.0896,	Borapetoside D or unknown isomer (C_33_H_46_O_16_)
12	13.1	**699.2866**	C_33_H_47_O_16_	1.0	[M+H]^+^	721.2676 (−0.3, [M+Na]^+^)	357.1694, 339.1584, 325.1453, 311.1644, 307.1348, 279.1375, 251.1426, 205.0860, 187.0742	Borapetoside D or unknown isomer (C_33_H_46_O_16_)
13	13.4	**537.2332**	C_27_H_37_O_11_	0.3	[M+H]^+^	554.2617 (3.8, [M+NH4]^+^), 559.2145 (−0.9, [M+Na]^+^)	357.1686, 339.1581, 325.1437, 307.1331, 297.1497, 279.1385, 251.1400	Borapetoside E or unknown isomer of borapetoside C or E (C_27_H_36_O_11_)
14	13.5	537.2338	C_27_H_37_O_11_	1.4	[M+H]^+^	559.2150 (−0.0, [M+Na]^+^),1095.4386 (−2.0, [2M+Na]^+^)	**357.1696**, 279.1366, 251.1451, 187.0756, 159.0809, 131.0849	Borapetoside E or unknown isomer of borapetoside C or E (C_27_H_36_O_11_)
15	13.7	**537.2331**	C_27_H_37_O_11_	0.1	[M+H]^+^	559.2148 (−0.4, [M+Na]^+^), 1073.4590 (0.2, [2M+H]^+^)	**375.1802**, **357.1699**, 339.1592, 325.1433, 311.1642, 307.1326, 297.1483, 279.1380, 251.1431, 205.0859, 187.0754, 159.0803	**Borapetoside C**
16	14.1	**681.2749**	C_33_H_45_O_15_	−0.6	[M+H]^+^	703.2571 (−0.2, [M+Na]^+^)	357.1696, 339.1559, 325.1426, 307.1344, 205.0851,	Unknown furanoditerpenoid structurally related to **1** (C_33_H_44_O_15_)
17	15.6	**343.1544**	C_20_H_23_O_5_	1.1	[M+H]^+^	365.1364 (1.2, [M+Na]^+^)	279.1400, 159.0817	Crispene A
18	16.7	375.1792	C_21_H_27_O_6_	−2.8	[M+H]^+^	397.1621 (−0.2, [M+Na]^+^), 771.3347 (−0.5, [2M+Na]^+^)	**357.1696**, 325.1430, 297.1491, 279.1385, 251.1426, 205.0855, 187.0753, 159.0806, 131.0850	Crispene D

Bolded values correspond to the masses labelling nodes in the cluster of borapetoside C in the molecular network generated with the data.

**Figure 5:**
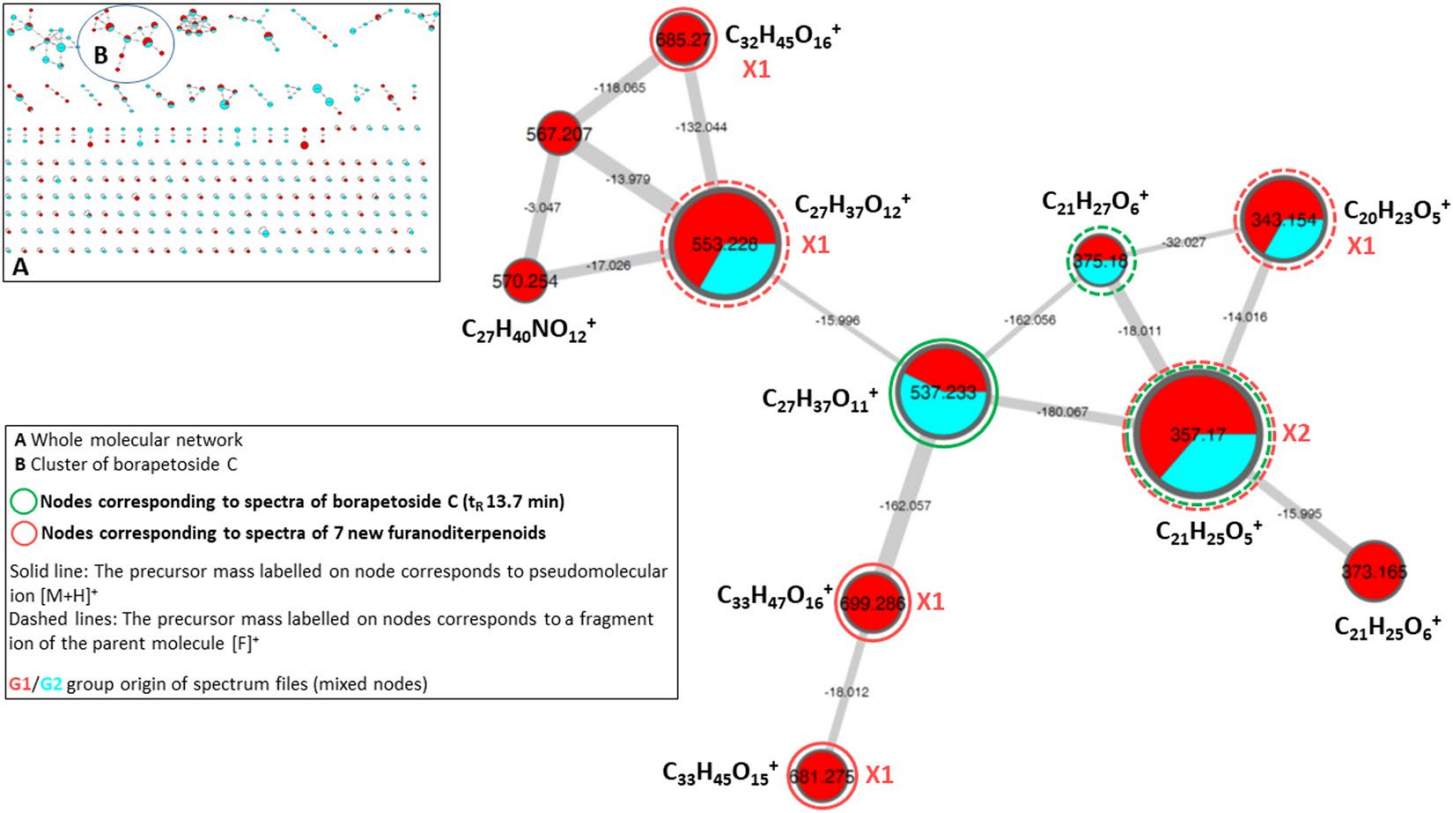
Molecular network generated with UHPLC-ESI-QTOF-HRMS^2^ data from the CH_2_Cl_2_ and aqueous extracts of *T. crispa* stems and visualized using Cytoscape software. Cosine similarity score cutoff was of 0.6. Nodes are labelled with parent or precursor mass value and their size are linked to the number of spectra (molecular formula of corresponding ions are indicated beside nodes). Nodes coloring: nodes are represented as pies and each color represents a group of spectrum files associated with an extract: red: G1/aqueous extract; blue: G2/CH_2_Cl_2_ extract. Edges are annotated with mass difference and their thickness depends on the cosine score ranging between 0.6 (minimum accepted similarity between spectra) and 1 (maximum similarity between spectra). See Material and Methods for supplementary details.

The molecular network generated with the MS/MS data acquired from both the CH_2_Cl_2_ and aqueous extracts is depicted in Fig. 5. Within the molecular network we focused our attention on the cluster exhibiting a central node associated with the precursor mass and the t_R_ of borapetoside C (**1**). It should be noted that under the adopted conditions of “one-shot” analysis (*i.e*. without use of enriched fractions and optimization of the collision energy) several nodes were found to correspond exclusively to fragment ion spectra and are thus labelled with the corresponding precursor mass. For instance, nodes labelled with the *m/z* values 375.180 and 357.170 correspond to fragment ion spectra of borapetoside C (**1**). However, to a single node may correspond several compounds either isomeric (*e.g. m/z* 685.270) or sharing a common fragment ion (*e.g. m/z* 553.228), then differentiated by their specific retention times. Finally, scrutinizing the cluster, in combination with complete inspection of MS/MS spectra and extracted ions chromatograms supported the presence of 17 additional furanoditerpenoid analogues of **1**, corresponding to peaks notified as No 1-14 and 16-18 in the base peak chromatogram (Fig. 3). Major fragment ions of these compounds are also listed in Table 2, as well as the parent ions values observed in the molecular network (bolded values).

**Figure 6:**
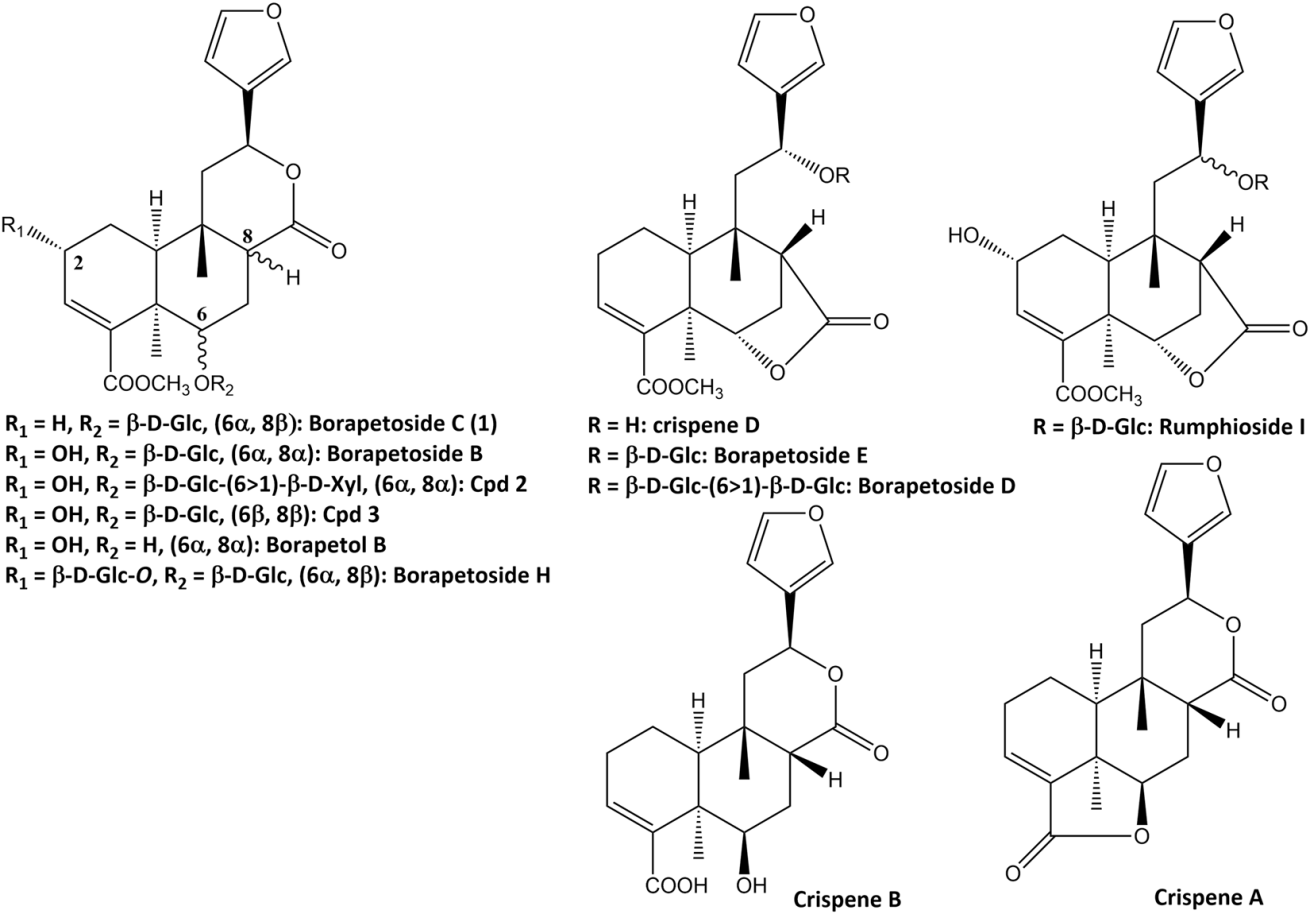
Chemical structures of furanoditerpenoids identified in *T. crispa s*tem aqueous extract.

Tentative identification of furanoditerpenoids analogues of **1** was based on the determination of molecular formulas deduced from accurate *m/z* values and fragmentation patterns and additionally through dereplication using database search including the Dictionary of Natural Products, Reaxys and SciFinder. Chemical structures of the putatively identified furanoditerpenoids are presented in Fig. 6.

Borapetoside C (**1**) showed a [M + H]^+^ ion at *m/z* 537.2331 (calculated for C_27_H_37_O_11_: 537.2330, Δppm = 0.3) and fragment ions at *m/z* 375.1802 and 357.1699, consistent with the loss of the 2-deoxyglucosyl (162 uma) and β-D-glucosyl (180 uma) moieties, respectively. Fragment ions produced by the successive elimination of methanol (32 uma) and carbon monoxide (28 uma) were characteristic of the presence of a carbomethoxy group (60 uma). For example, the fragment ions observed at *m/z* 325.1433 and 297.1483 may be formed from the protonated ion at *m/z* 357.1699 as well as the fragment ion at *m/z* 251.1431 may be produced from the cationic ion at *m/z* 311.1642. The concomitant neutral loss of CO_2_ and C_6_H_6_O (attributed to 3-vinylfuran), accounting for 138 uma, was typical fragmentation pattern of the 6-furyl-tetrahydro-2*H*-pyran-2-one structure and may explain the formation of fragment ions observed at *m/z* 205.0859, 187.0754 and 159.0803.

Compounds eluting at 11.8, 13.0, 13.1, 13.4, 13.5, 14.1, 15.6 and 16.7 min (peaks 9, 11–14, 16–18, respectively) and borapetoside C (**1**) shared the same fragmentation patterns and should consequently display a close similarity in their structures.

Compounds corresponding to peaks 13 and 14 exhibited the same pseudomolecular ion at *m/z* 537.2332/537.2338, respectively (calculated for C_27_H_37_O_11_: 537.2330, Δppm = 0.3/1.4) as that of **1** and both could be tentatively assigned to borapetoside E^10,11^ or to unknown stereoisomers of borapetosides C and E. Peaks 9, 11 and 12, could be tentatively assigned to borapetoside D (=6′ → 1″)-*O*-β-D-glucopyranosyl borapetoside E)^10,11^ or to unknown isomers of this molecule including *O*-hexosyl borapetoside C derivatives. They exhibited a same protonated ion at *m/z* 699.2842/699.2859/699.2866, respectively (calculated for C_33_H_47_O_16_: 699.2859, Δppm = −2.4/0.0/1.0). Peaks No 17 (t_R_ of 15.6 min) and 18 (t_R_ of 16.7 min) may correspond to crispenes A and D^12^, respectively. These compounds showed a [M + H]^+^ ion at *m/z* 343.1544 (calculated for C_20_H_23_O_5_: 343.1540, Δppm = 1.1) and 375.1792 (calculated for C_21_H_27_O_6_: 375.1802, Δppm = −2.8), respectively. Finally, the compound corresponding to peak No 16 (t_R_ of 14.1 min) displayed a protonated ion at *m/z* 681.2749 (calculated for C_33_H_45_O_15_: 681.2753, Δppm = −0.6) and has not been previously reported. The mass difference observed between the protonated ions of this compound and that of **1** was 145 uma, which could be reasonably attributed to one additional ester group of a hydroxylated dicarboxylic acid (C_6_H_10_O_5_).

**Figure 7:**
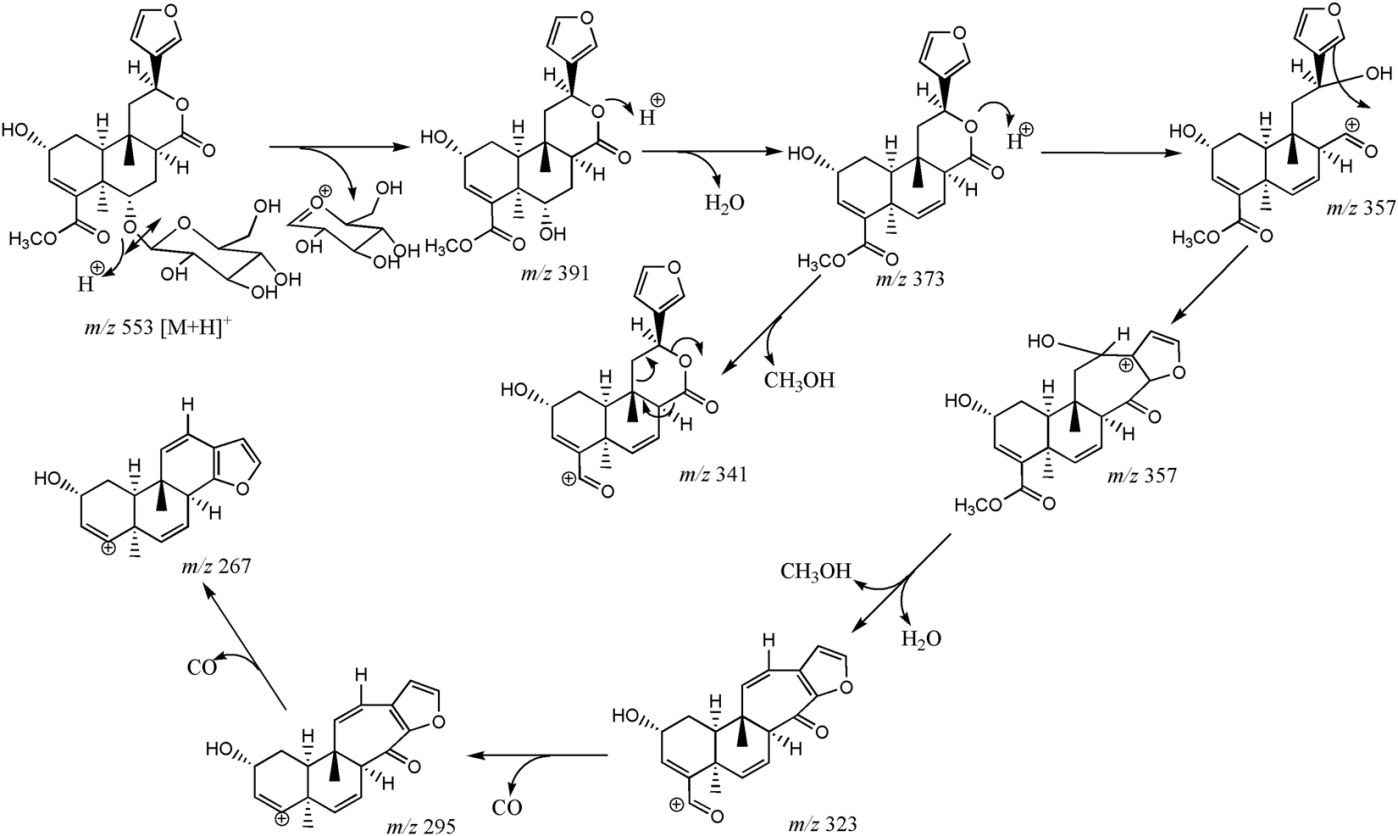
Proposed fragmentation pathways of borapetoside B (**1**).

A second group of borapetoside C analogues is represented by compounds bearing a hydroxyl group at the C-2 position such as borapetoside B. They all shared common fragmentation pathways with probably similar reactions to those described for **1**, as illustrated in Fig. 7 for borapetoside B. However, protonated parent ions and fragment ions displayed a characteristic difference of mass of 16 uma attributed to oxygen in comparison to analogous ions observed in the tandem mass spectra of **1**. Compounds belonging to this group are significantly more hydrophilic and were eluted between 7.0 and 12.0 min (peaks 1–6 and 10).

Compounds corresponding to peaks 5 (t_R_ of 9.1 min) and 6 (t_R_ of 9.3 min) exhibited the same pseudomolecular ion at m/z 553.2277/553.2279, respectively (calculated for C_27_H_37_O_12_: 553.2280, Δppm = −0.5/−0.1) and both could be tentatively assigned to borapetoside B^8^ or its reported isomers (*i.e*. rumphioside I^11^ and the diastereosiomer **3**^13^). Peak No 1 (t_R_ of 7.0 min) may correspond to borapetoside H^2^ since it displayed a protonated parent ion at *m/z* 715.2828 (calculated for C_33_H_47_O_17_: 715.2810, Δppm = 2.8). Peaks No 3 (t_R_ of 8.2 min) and 4 (t_R_ of 8.7 min) displayed a same [M + H]^+^ ion at *m/z* 685.2687/685.2700, respectively (calculated for C_32_H_45_O_16_: 685.2702, Δppm = −2.2/−0.3) and both may be tentatively assigned as the reported (2*R*,5*R*,6*R*,8*S*,9*S*,10*S*,12*S*)-15,16-epoxy-2-hydroxy-6-*O*-{β-D-glucopyranosyl-(1 → 6)-α-D-xylopyranosyl}-cleroda-3,13(16),14-trien-17,12-olid-18-oic acid methyl ester (**2**)^13^ or an unknown isomer of this compound. Peak No 10 (t_R_ of 12.0 min) could be tentatively identified as borapetol B^10^, the aglycon moiety of borapetoside B. This compound exhibited a [M + Na]^+^ at *m/z* 413.1570 (calculated for C_21_H_26_NaO_7_: 413.1571, Δppm = −0.2). Compound eluted at t_R_ = 7.9 min (peak No 2) showed a [M + Na]^+^ at *m/z* 593.2209 (calculated for C_27_H_38_NaO_13_: 593.2205, Δppm = −1.2). This new compound is most likely the corresponding analogue of either borapetoside B or its isomers, with the lactone ring opened and converted to 5-hydroxypentanoic acid moiety.

A last group corresponds to compounds eluted at 9.7 (peak No 7) and 11.7 min (peak No 8). These compounds shared some fragment ions with borapetoside C (**1**) and its close structural analogues particularly those at *m/z* 251, 159 and 131, but also exhibited in their MS/MS tandem spectra several specific fragment ions. Peak No 8 was tentatively assigned to the non-glycosidic furanoditerpenoid crispene B^12^, characterized by the presence of a carboxyl group at C-4 in place of a carbomethoxyl group. This compound showed a [M + Na]^+^ at *m/z* 383.1465 (calculated for C_20_H_24_NaO_6_: 383.1465, Δppm = −0.1) and a characteristic protonated fragment ion at *m/z* 343.1543 which corresponds to the loss of H_2_O ([M-H_2_O+H]^+^). Compound eluting at 9.7 min exhibited quasimolecular molecular ions at *m/z* 545.1998 ([M + Na]^+^: calculated for C_26_H_34_NaO_11_: 545.1993, Δppm = + 0.9) and at *m/z* 567.1804, which corresponds to a deprotonated carboxyl group ([M-H + 2Na]^+^: calculated for C_26_H_33_Na_2_O_11_: 567.1813, Δppm = −1.5), as well as fragment ions at *m/z* 383.1468 ([M-162 + Na]^+^), 365.1358 ([M-180 + Na]^+^) and 343.1541 [M-180 + H]^+^. We concluded from these data that this compound was an unknown *O*-glycosyl derivative of crispene B or of its isomers (such as, for example, the corresponding carboxylic acid of **1**).

Interestingly, apart from mass and chromatographic information provided by molecular network exploration, node coloring indicates that all these compounds were detected in the aqueous extract. This observation is consistent with the presence of highly polar/hydrophilic molecules such as, for instance, borapetosides D and H which bear two sugar units, as well as borapetoside B and rumphioside I with a free hydroxyl group at the C2 position.

## Discussion

Among the large spectrum of traditional use of *T. crispa* reported in the literature, its anti-diabetic activity has raised a special interest for researchers all around the world considering the growing number of type 2 diabetes cases, all the more so as its use in South-Asia has been widespread for a long time. We have discovered that the plant is also consumed in the French West Indies (and probably throughout the Caribbean Arc due to the historic intermingling of Caribbean populations). Indeed, a survey has shown that 60% of diabetic patients in Martinique (French West Indies) use plants in addition to their glucose lowering drugs, among them *T. crispa* was the second plant the most used^14^. However, like several alternative medicine therapies for diabetes, finding reliable information about safety and benefits remains difficult. Notably, adverse effects associated with traditional medicine are often not well documented.

In this report, we present a case of toxic hepatitis following occasional consumption of a stem aqueous extract of the *T. crispa*. Hepatitis induced by *T. crispa* is established, based on absence of medical history, clinical exam and biological results. We noted that the patient had been exposed to several pesticides the next day after the last ingestion of the aqueous extract. Yet, to the best of our knowledge acute cutaneous or respiratory exposure to those pesticides (containing myclobutanil, glyphosate, acetamipride, abamectin and linuron) could not explain the onset of hepatitis. Hepatitis was reversible and biological hepatic enzymes returned to normal after a few weeks without any specific treatment. This conclusion was corroborated by calculation of Roussel Uclaf Causality Assessment Method (RUCAM) score. With a calculated score of +6, the causal relationship between *T.c*. aqueous extract intake and hepatotoxicity was “probable”^15^.

Two previous cases of acute hepatitis have been reported with *T. crispa* but with a different method of administration consisting of chronic use of tablets or pellets of the plant. The first case of hepatitis concerned a 37-year-old woman who had consumed *T. crispa* tablets (bought in Indonesia) for 10 weeks^3^. The second case concerned a 49-year-old man who had orally taken pellets of *T.crispa* (bought on a Vietnamese market^4^) for 4 weeks. In both cases, hepatitis was reversible after a few weeks. These case reports are in accordance with changes in biochemical parameters observed during some clinical trials assessing the effect of *T. crispa* in diabetic patients and in patients with metabolic syndrome. Marked elevation of liver enzymes (that returned to normal after discontinuing *T. crispa*) has been observed in 2 of the 20 patients treated for 6 months with a capsule form at a dosage of 1 gram thrice daily^16^. Similarly, a double-blind, placebo-controlled trial using a crossover design found an elevation of more than 3 times baseline levels of ALT and AST in 6 of the 36 patients who received 250 mg *T. crispa* dry powder capsule twice a day for 2 months^17^.

We hypothesize that two mechanisms may have contributed to hepatotoxicity induced by *T. crispa*, considering the close structural similarity observed between borapetosides and i. furanoditerpenoids like 8-Epidiosbulbin E acetate (EEA) and ii. teucrin A or teuchmaedryn A. The first mechanism is direct liver toxicity induced by metabolic activation of *T. crispa* furanoditerpenoids. Such a mechanism of toxicity has been described with EEA, a norclerodane furanoditerpenoid structurally very similar to borapetosides^18^. Cytochrome P450 metabolic activation of the furan moiety of EEA may be responsible for the formation of electrophilic species, leading to a dose-dependent hepatotoxicity. Other similar examples of bioactivation of furanoditerpenoids correlated with hepatotoxicity are reported in literature^18,19^. The second toxicological mechanism may be idiosyncratic, as it has been reported that only a few people exposed may develop hepatitis^16,17^. Such a mechanism may be suspected in our case, as hepatitis occurred after reexposure to *T. crispa* and was associated with fever, a typical symptom of idiosyncratic immunoallergic toxic hepatitis. Moreover, there is a close structural analogy between borapetosides and teucrin A or teuchmaedryn A, two clerodanes found in *Teucrium chamaedrys* L. (Lamiaceae). *In-vivo* and *in-vitro* studies have demonstrated that those teucrin A or teuchmaedryn A induce hepatotoxicity, consecutive to both direct toxicity and a secondary immune reaction with antoantibody formation^20^. Future studies using isolated compounds will be necessary to ascertain the exact mechanism of toxicity of the furanoditerpenoids from *Tinospora crispa* stems.

The present UHPLC-ESI-QTOF-MS/MS metabolomic investigation performed on the aqueous stem extract of *T. crispa* led to the detection of 18 furanoditerpenoids structurally related to the major compound, namely borapetoside C (**1**). A targeted analysis of MS/MS data was facilitated by introducing a molecular networking approach in the data analysis workflow. MN is becoming one of the most efficient tools for the analysis of untargeted MS/MS data, allowing dereplication of complex extracts and exploration of molecular diversity^21^. We have showed here that MN is also valuable for targeted metabolite fingerprinting. To the best of our knowledge this is the first example of application of MN for investigating plant suspected of being toxic and toxins thereof.

Hepatic trouble should be put in perspective with the potential hypoglycaemic effect of the plant. Promising results have been obtained by *in vitro* and *in vivo* tests with *T. crispa*. Noor *et al*. have observed insulin secretory rates in alloxan-diabetic rats and insulin release from rat islets and HIT-T15 beta cells *in vitro* with the extract of stem plant^22^. Since this publication, its hypoglycaemic mechanism of action has been attributed to diterpenoids (borapetol B, borapetoside A and C) isolated from the plant that could stimulate insulin release from pancreatic β-cell. Using an oral glucose tolerance test, an increase of 2-fold of plasma insulin release has been observed after oral administration of borapetol B to Wistar rats and spontaneously type 2 diabetic Goto-Kakizaki rats compared to placebo^23^. Similarly, administration of borapetoside A to diet-induced type 2 diabetes mellitus mice lowered the plasma glucose level in a dose-depended manner with results similar to the administration of metformin for the dose range between 0.3 and 10 mg/kg^24^. A potential mechanism of improvement of peripheral glucose uptake, notably via an increase in glucose utilization of skeletal muscle and liver, has also been advanced following *in vitro* tests^24–26^. To date, these promising non-clinical studies have unfortunately not been confirmed by clinical trials applied especially to type 2 diabetic patients. Indeed, Klangjareonchai *et al*. showed that glucose and insulin areas under the curve were not different with or without ingestion of 125 or 250 g of *T. crispa* dry power capsule^27^, while Sangsuwan *et al*. in a randomized, double blind, placebo-controlled trial found no difference on HbA1c or fasting plasma glucose between patients treated with 1 gram thrice daily *Tinospora crispa* powder in capsule for 6 months and patients treated with placebo^16^.

In total, the Benefit-Risk of the use of *Tinospora crispa* for the treatment of type 2 diabetes appears negative at this step, despite the hope raised by non-clinical studies. There remain uncertainties on the efficacy on blood glucose control in real-life setting while the risk of hepatic trouble is established, both for an acute and for a chronic use and independently of the method of administration (stem in powder capsule or used as an aqueous extract). Furthermore, toxic mechanisms may associate both dose-response relationship and idiosyncratic effects, therefore making therapeutic use of *T. crispa* hazardous, in so far as diabetic patients are often treated simultaneously with statins, drugs known to potentially increase liver enzymes.

## Conclusion

Chronic or occasional use of *T. crispa* stem could induce toxic hepatitis, reversible after a few weeks without any specific treatment. Despite its promising results suggesting an increase in insulin release in non-clinical studies, its traditional use should be avoided for diabetic patients for lack of demonstrated benefit obtained by dedicated well-controlled clinical trials. Several furanoditerpenoids have been detected and putatively identified by UHPLC-ESI-QTOF-MS/MS in an aqueous extract of fresh stems that mimics the traditional preparation by combining classical data exploration and molecular networking approach. Even in non-optimized conditions of data acquisition, molecular networking constitutes a powerful and useful tool facilitating the data filtering. Further studies are still needed to confirm the putative toxicity of furanoditerpenoids and to elucidate subjacent mechanisms.

## Materials and Methods

### Questioning and biochemical analyses

Informed consent was obtained from the patient. The Faculté de Pharmacie de Paris, Université Paris Descartes approved the experimental protocol and it was carried out in accordance with all relevant guidelines and regulations. Biochemical analyses were performed by standard methods using automated techniques.

### Standards

#### Authentic herbal reference standard

Stems were originally collected in 2013 in the Botanical Garden of the Hanoi University of Pharmacy in Hanoi, Vietnam and authenticated par Plant Records Officer Dr Quoc Huy Nguyen. A voucher specimen is stored in the herbarium collection of the François Tillequin Museum of Materia Medica (Faculty of Pharmacy, Paris) under the N°02486. *Borapetoside C*. Borapetoside C was isolated from *T crispa* in our laboratory by column chromatography and unambiguously identified by ^1^H, ^13^NMR and MS by comparison with literature data^9,10^.

### Sample preparation

#### Dichloromethane extracts of *T. crispa* stem

Test and reference solutions were prepared in the same way by maceration of stems in methylene chloride for 72 h, followed by filtration, then drying under vacuum (Drug Extract Ratio (DER): about 47:1 and 24:1, respectively). *Aqueous extracts of* T. crispa *stem*. Given the limited amount of material available (*i.e*. 1.2 g) stems were boiled in water (10 mL) for 10 min and the resulting solution was filtered prior to lyophilisation (DER: about 100:1). Moreover, the complete solubility of the lyophylisate in deionized water at ambient temperature (20 °C) was checked at a concentration of 5.6 mg/mL.

### Chromatographic conditions

#### RP-HPLC-UV (ESI-MS) conditions

HPLC-System: Thermo Scientific Dionex Ultimate 3000®, Courtaboeuf, France) with binary pump, PDA detector, autosampler and Chromeleon software. Detection: 214 nm. Injection volume: 20 μL. Column: Zorbax SB®-C18 (5 μm, 4.6 × 250 mm), equipped with a pre-column and thermostated at 25 °C. Mobile Phase: Gradient: [MeOH:Water-0.05% TFA]: 10:90 → 80:20 within 60 min). Flow: 0.6 mL/min. All solutions were prepared in MeOH (2 mg/mL) and filtered through 0.2 μm nylon filter disk prior the injection. ESI-MS spectrum (Thermo-Scientific Surveyor MSQ Plus single-quadrupole mass detector (Thermo Fisher Scientific, Courtaboeuf, France)).

#### UHPLC-HRMS and MS^2^ experiments

Separations were performed using an Ultimate 3000 RSLC system equipped with a binary pump, an autosampler and a thermostated column compartment, equipped with a diode array detector (195–800 nm) (Dionex, Germering, Germany). Components were separated on a C18 Luna Omega column of 150 mm × 2.1 mm with a particle size of 1.6 μm (Phenomenex, Le Pecq, France). The mobile phase was made up of 0.1% formic acid in water (phase A), and 0.08% formic acid in acetonitrile (phase B). A solvent gradient was applied as follows: 0–0.1 min: 3% B, 0.1–26 min: 3–80% B, 26–26.5 min: 80–95% B, 26.5–29.5 min: 95% B, 29.5–30 min: 95–3% B, and finally 30–33 min: 3% B. The column was introduced in a thermostated compartment heated at 40 °C. All solutions were prepared in MeOH (2 mg/mL) and filtered through 0.2 μm nylon filter disk prior the injection. The injection volume was set at 1 µL and the flow rate was set at 500 μL/min. MS experiments were carried out on a maXis UHR-Q-TOF mass spectrometer (Bruker, Bremen, Germany) in positive electrospray ionization (ESI) mode. Capillary voltage was set at +4.5 kV. The flows of nebulizing and drying gas (nitrogen) were respectively set at 2.0 bar and 9.0 L/min and drying gas was heated at 200 °C. Mass spectra were recorded in the range 50–1650 *m/z*. MS/MS experiments were conducted using data dependent acquisition (DDA) mode (auto-MS/MS) in a mass window from *m/z* 150–1200. Three precursor ions with intensities higher than 400 au were selected per fragmentation cycle among the most intense ions to be fragmented. These three precursor ions were allowed to be selected for two consecutive cycles and were then placed on an exclusion list for 0.05 min. The collision energy was set at 35 eV and was applied as follows: 88% of the collision energy was applied during half of the fragmentation cycle, and 117% of the collision energy was applied during the half remaining cycle time. Raw data were converted to mzXML format using CompassXport software (Bruker, Bremen, Germany) and then processed using MZmine version 2^28^.

#### Molecular Network Analysis

A molecular network was created using the online workflow at GNPS (https://gnps.ucsd.edu/ProteoSAFe/static/gnps-splash.jsp)^6^. The data was filtered by removing all MS/MS peaks within +/−17 Da of the precursor *m/z*. MS/MS spectra were window filtered by choosing only the top 6 peaks in the +/−50 Da window throughout the spectrum. The data was then clustered with MS-Cluster with a parent mass tolerance of 0.05 Da and a MS/MS fragment ion tolerance of 0.05 Da to create consensus spectra^29^. Further, consensus spectra that contained less than 1 spectrum were discarded. A network was then created where edges were filtered to have a cosine score above 0.6 and more than 2 matched peaks. Further edges between two nodes were kept in the network if and only if each of the nodes appeared in each other’s respective top 20 most similar nodes. The spectra in the network were then searched against GNPS’ spectral libraries. The library spectra were filtered in the same manner as the input data. All matches kept between network spectra and library spectra were required to have a score above 0.7 and at least 6 matched peaks. Molecular network parameters are available at https://gnps.ucsd.edu/ProteoSAFe/status.jsp?task=2817c0f504f741f781f645eb3ee27d95.

## Data Availability

Data of LC-MS/MS analysis were deposited to MassIVE Public GNPS dataset (http://gnps.ucsd.edu/MSV000082664).
